# Symptoms and Quality of Life in Late Stage Parkinson Syndromes: A Longitudinal Community Study of Predictive Factors

**DOI:** 10.1371/journal.pone.0046327

**Published:** 2012-11-07

**Authors:** Irene J. Higginson, Wei Gao, Tariq Zaffer Saleem, K. Ray Chaudhuri, Rachel Burman, Paul McCrone, Peter Nigel Leigh

**Affiliations:** 1 Cicely Saunders Institute, King's College London, London, United Kingdom; 2 Institute of Psychiatry, King's College London, London, United Kingdom; 3 National Parkinson Foundation International Centre of Excellence, Kings College Hospital, London, United Kingdom; Oslo University Hospital, Norway

## Abstract

**Background:**

Palliative care is increasingly offered earlier in the cancer trajectory but rarely in Idiopathic Parkinson's Disease(IPD), Progressive Supranuclear Palsy(PSP) or Multiple System Atrophy(MSA). There is little longitudinal data of people with late stage disease to understand levels of need. We aimed to determine how symptoms and quality of life of these patients change over time; and what demographic and clinical factors predicted changes.

**Methods:**

We recruited 82 patients into a longitudinal study, consenting patients with a diagnosis of IPD, MSA or PSP, stages 3–5 Hoehn and Yahr(H&Y). At baseline and then on up to 3 occasions over one year, we collected self-reported demographic, clinical, symptom, palliative and quality of life data, using Parkinson's specific and generic validated scales, including the Palliative care Outcome Scale (POS). We tested for predictors using multivariable analysis, adjusting for confounders.

**Findings:**

Over two thirds of patients had severe disability, over one third being wheelchair-bound/bedridden. Symptoms were highly prevalent in all conditions - mean (SD) of 10.6(4.0) symptoms. More than 50% of the MSA and PSP patients died over the year. Over the year, half of the patients showed either an upward (worsening, 24/60) or fluctuant (8/60) trajectory for POS and symptoms. The strongest predictors of higher levels of symptoms at the end of follow-up were initial scores on POS (AOR 1.30; 95%CI:1.05–1.60) and being male (AOR 5.18; 95% CI 1.17 to 22.92), both were more predictive than initial H&Y scores.

**Interpretation:**

The findings point to profound and complex mix of non-motor and motor symptoms in patients with late stage IPD, MSA and PSP. Symptoms are not resolved and half of the patients deteriorate. Palliative problems are predictive of future symptoms, suggesting that an early palliative assessment might help screen for those in need of earlier intervention.

## Introduction

Idiopathic Parkinson's Disease(IPD), Progressive Supranuclear Palsy(PSP) and Multiple System Atrophy(MSA) share many symptoms and signs in common, have profound motor and non-motor symptoms and effects on quality of life, health care and personal costs [1]–[5]. Symptoms include instability, fatigue, dribbling, pain, psychological distress, cognitive impairment and their prevalence correlates with Hoehn and Yahr stage [1], [6]–[8]. However, little is known about how symptoms, quality of life and impacts evolve in the later stages of these disorders.

We searched PubMed for articles using the keywords: (late stage OR advanced OR palliative OR terminal care OR end of life) AND (Parkinson's OR MSA OR PSP) AND (longitudinal OR cohort) AND (quality of life OR Symptoms OR psychological distress).

Longitudinal data are rare and focussed on mild to moderate disease often during treatments [1], [6], [7], [9]–[11]. The NNIPPS study, a randomised trial of Riluzole in MSA and PSP provides valuable information on the progression of disease, survival, cognition and some symptoms [5], [8], [12], [13], but not the wide range of motor and non-motor problems that can be experienced in late stage disease [14]. Palliative care is offered to cancer patients early in the disease course and to people affected by amyotrophic lateral sclerosis in later stages of illness [15]–[17]. In contrast, few patients with even late stage IPD, PSP or MSA have access to palliative care or hospice services [17]. Lack of information about the trajectory of symptoms and quality of life in late stage IPD, PSP and MSA means that it is not clear: (a) whether patients' and care-givers' needs are adequately met by existing services or whether augmentation by specialist palliative care services would be helpful; and (b) if the latter is the case, what would be the optimal strategy for referral in relation to patient and care-giver needs and disease evolution? Cross sectional studies of patients at different stages can be biased and oversimplify the course of illness [18], [19]. Thus longitudinal studies that consider individual trajectories are likely to provide more accurate insights into patient needs and factors that might predict likely needs for referral.

In this study we sought to answer two questions: a) how do symptoms (especially those common in palliative care and at the end of life in other conditions, such as pain, breathlessness, fatigue, psychological distress) [20], [21] and quality of life of Parkinson syndrome patients change over time; and b) what demographic and clinical factors predict these changes. The unique nature of this study is the focus on palliative care needs, late stage disease and home settings.

## Methods

We conducted a longitudinal study over one year in community settings. The study was approved by the Research Ethics Committee of the Institute of Psychiatry and the Research Ethics Committee of King's College Hospital NHS Foundation Trust.

The study was conducted according to the principles expressed in the Declaration of Helsinki and all procedures were approved by both the Research Ethics Committee of the Institute of Psychiatry and the Research Ethics Committee of King's College Hospital NHS Foundation Trust. All participants were given full information regarding the study, signed to indicate their consent to take part, and could withdraw at any time. Participation, withdrawal or not, had no effect on the health care or other services received. All comments and information was kept confidential, and patient identifying information was not recorded on the questionnaires, but kept separately from their consent forms.

### Participants

Patients were recruited from those attending King's College Hospital outpatient specialist neurology movement disorder clinic, a secondary and tertiary clinic service with a geographical catchment area of 4000 IPD patients altogether, covering South East England (London, Kent, Sussex).

We recruited consenting patients attending the clinic with a clinical diagnosis of IPD, MSA or PSP, who fell into stages 3–5 of the Hoehn and Yahr system [22] for IPD and equivalent motor disability for MSA and PSP. Patients were excluded if they were already resident in nursing homes. When possible patients' nearest lay caregivers were also recruited.

### Procedures for Baseline and Follow-up Interviews

Following recruitment we arranged baseline face to face interviews in patients' place of choice, usually their home. We also interviewed the nearest caregiver or family members, in the home or place of choice. Three follow-up interviews were organised over the course of 12–15 months, aiming to conduct interviews at 3–4 monthly intervals. Interviews invariably involved travelling to patients homes, but we believed this would be necessary for subjects with profound disability. We kept in touch with patients by telephone and checked their condition with clinical services before re-contacting them. Repeat visits were agreed if, on arrival, the subject did not feel up to some of, or the entire, interview. Patients were followed if they entered nursing or care homes, hospices or hospital. Where possible we sought information about any deaths that occurred. All interviews were conducted by one of three centrally trained researchers with psychology or palliative nursing backgrounds. The researchers used a standard questionnaire to capture patients' self-reported demographic and clinical characteristics, the Hoehn and Yahr(H&Y) classification (both as assessed and verified by checking the clinical records), widely used to establish the staging of IPD(21), symptoms and quality of life. Interviews took 1–2 hours in the home; sometimes longer if the patients wanted to take a break or had a lot to say. On occasions we returned for interview, as on arrival it was not found to be a convenient time, or to interview the caregiver. If patients had considerable difficulties or distress when visited, the researcher followed a distress protocol, to check that the patient/caregiver were happy to continue with the interview and whether they wanted the researcher to make contact with services for them. On a small number of occasions, with the patient's consent, the researcher contacted services on their behalf for symptom problems or missed follow-up.

### Clinical, Symptom, Psychological and Quality of Life Assessments

Table 1 shows the measures administered at all time points. Parkinson's specific (PDQ-8) and generic measures (HADS-14, EQ5D, POS, POS-PP) were used.

**Table 1:** The Parkinson's specific and generic quality of life, symptom and palliative care measures administered at all time points.

Measure	Number of items, content and scoring	Validation/references
Parkinson's Disease Questionnaire(PDQ)-8	8 Items. Each item is rated: 0 (never) to 4(always/cannot do) in the past one month. Items are: problems getting around in public, difficulty dressing self, felt depressed, embarrassed in public due to having disease, problems with close personal relationships, problems with concentration, unable to communicate with people properly, painful muscle cramps or spasms. PDQ-8 Summary Index is expressed as percentage of the sum of the items scores on the maximum possible scale score [33].	Extensively validated in IPD and related disorders, including translated versions and in different cultures, provides a reliable measure of overall health status recommended for studies in which a short questionnaire is preferred [34]–[36]. It maps and shows a similar responsiveness to change as the EQ5-D [33], [37], [38].
Hospital Anxiety and Depression Scale(HADS)-14	14 items: 7 concerned with anxiety and 7 with depression. Each item is rated on a 0–3 (worst) scale. Total possible score is 42 (worst psychological distress).	HADS has been used widely in Parkinson syndromes and many diseases. There are two subscales – depression and anxiety but Rasch analysis in patients with Parkinson's Disease supports the use of total score to assess psychological distress [39], [40].
EQ5-D	5+2 items. EQ5-D has three components: 1) a descriptive part, consisting of 5 items, that can then be converted into a value (EQ-Index), each ranging from 1 (perfect health state) to 0 (death); 2) a question about change in health status in the preceding 12 months; and 3) a visual analogue scale (EQ-VAS) for assessment of current health state, from 0, the worse imaginable health state, to 100, best imaginable health state.	ED5-D is a generic widely used HRQoL measure. It has been used widely in IPD [37], [38], [41]–[43]. EQ5-D was calculated using the UK weighting schemes recommended by Dolan P et al [44].
Palliative care Outcome Scale (POS)	10 items. Each item is rated according to how much it has affected the person, from 0 (best) to 4 (overwhelming problem) over the past 2 weeks. Items are: pain control, symptom control, patient anxiety, family anxiety, information, sharing feelings, depression, self-worth, practical needs and time wasted. Total possible score 40.	POS is one of the top two outcome measures used in palliative care studies, has psychometric and clinimetric validity, reliability and responsiveness to change, in late stage and earlier but symptomatic disease across many conditions and settings, including neurological conditions (multiple sclerosis, motor neurone disease), cancer, organ (respiratory, renal and heart) failure, elderly patients with co-morbid disease, in home, hospital, nursing home, hospice settings. Research has shown it can be used as an overall scale, as individual items or 3 factors; physical, quality of care and psychological [23], [45]–[48].
Palliative care Outcome Scale for Symptoms (POS-PP)	20 items (motor and non-motor symptoms). Each item is rated according to how much it has affected the person, from 0 (best) to 4 (overwhelming problem) over the past 2 weeks. Total possible score of 80.	A validated extension of the core POS assessing symptoms (POS-S), with additional Parkinsonism Plus symptoms added. POS-S measure has been used in many conditions, including neurological diseases [24], [45], and POS-PP is essentially POS-S with some specific Parkinson's related symptoms added. There are no generic palliative care measures that have been used and validated in IPD, MSA or PSP; therefore POS and POS-PP because of their proven validity in late stage disease and simplicity of use were the best fit.

### Outcome and Predictor Variables

Outcome were total scores on the POS-PP, the core POS (our primary outcomes), the PDQ-8 and the HADs-14 at follow-up interviews. Predictor variables were categorised into socio-demographic (age, sex, ethnic group, employment status, education, living arrangement); clinical and health care related variables (informal carer relationship, next of kin, disease type, stage of disease, follow-up status, median time in study), and baseline scores on the POS-PP, the core POS, the PDQ-8 and the HADs-14. We also created four new outcome variables (ind_POS-PP, ind_core-POS, ind_PDQ-8, ind_HADs-14). The values of these new variables were determined by counting the number of follow-up scores higher than the mean baseline score for that measure. The new variables were in ordinal format with a value range of 0 to 3.

### Statistical Analysis

Data were reported as means (SDs) or medians (ranges) if continuous, and count (percent) if categorical. The group trajectories of the total scores over time (the follow-up interviews) were plotted for the POS-PP, the POS, the PDQ-8 and the HADs-14. Ordinal logistic regression with the proportional odds assumption was used to identify potential predictors of the high follow-up scores. We first tested the bivariate association between the new outcome variables (categorised into final outcome worse than or better than the mean) and potential predictors (including baseline scores of four instruments, and demographic and clinical variables) using ordinal regression. Those predictors found to be significant at the 0.2 level in bivariate analyses were used to build multivariable models and to adjust for confounding effect between predictors. Adjusted risk ratios (95% CI) were then estimated from the multivariable models. We did not test the interaction effects due to the small sample size. Analyses were performed for four outcome variables separately. The main analyses included non-missing data only. Analyses were repeated on multiple imputed data. Sample size calculation was based on a mean difference of 0.5 (SD = 1) in the primary outcome measures (POS-PP, Core-POS) among subgroups or over time. We need a minimum 60 patients for a two-sided test detecting such difference with 80% power and at a 5% significance level. Assuming prevalence of symptoms in this patient population to be around 50%, 45 patients would allow us to achieve a precision of 7% in the estimation of symptom prevalence. Allowing for attrition at 25–30% over the course of one year this would mean we needed to recruit 80–85 patients into the study. All statistical analyses were completed by using SAS version 9.2 (SAS Institute, Inc., 2000 Cary, NC) and a two-sided 0.05 was considered significant.

## Results

### Demographic and clinical characteristics

A total of 82 patients consented and were recruited into the study over a period of 30 months (Table 2). Median disease duration (range) was 8.0 years (1.0–25.0) from diagnosis [IPD: 5.8(1.0–25.0), MSA: 4.0(1.0–14.0), PSP: 2.7(1.0–11.0) years]. Over two thirds of patients had at least severe disability – with over one third being wheelchair-bound or bedridden; disability was higher for PSP and MSA than IPD. Most patients (79) were unable to work due to their illness, 65 had retired and only 4 were in current part-time employment. Spouses were the most common carers – 35 patients had wife/partners and 24 had husband/partners who acted as informal carers, all lived with their spouse/partner. 14 patients lived alone, of whom four had no informal carer although formal care was in place for these patients.

**Table 2:** Characteristics of the 82 patients, total and by diagnosis: IPD, MSA, PSP.

Characteristics	Value	IPD	PSP	MSA	Total
All	Total, n(%)	50(61%)	15(18%)	17(21%)	82(100%)
Gender	Men n(%)	27(54%)	7(47%)	11(65%)	45(55%)
Age	Mean(SD)	67(8)	69(11)	67(8)	67(9)
	Median(min, max)	68(40, 80)	71(38, 86)	68(51,81)	68(38,86)
Ethnic group	White UK	35(70%)	12(78%)	17(100%)	64(78%)
	White Irish	2(4%)	0(0%)	0(0%)	2(2%)
	South Asian	6(12%)	1(7%)	0(0%)	7(8%)
	Black Caribbean	3(6%)	0(0%)	0(0%)	3(4%)
	Chinese	1(2%)	0(0%)	0(0%)	1(1%)
	Other	3(6%)	2(12.5%)	0(0%)	5(6%)
Employment	Retired	38(76)	12(80%)	11(65%)	61(74%)
	In employment or self-employed	6(12%)	0(0%)	0(0%)	6(7%)
	Unable to work (due to illness)	6(12%)	3(20%)	6(35%)	15(18%)
Hoehn & Yahr*	3	20(40%)	2(13%)	2(12%)	24(29%)
	4	19(38%)	4(26%)	7(41%)	30(37%)
	5	11(22%)	9(60%)	8(47%)	28(34%)
Informal carer	No informal carer	7(14%)	1(7%)	1(6%)	9(11%)
	Spouse/Partner	36(72%)	11(73%)	12(71%)	59(72%)
	Daughter	4(8%)	2(13%)	2(12%)	8(10%)
	Sibling	2(4%)	1(7%)	1(6%)	4(5%)
	All others	1(2%)	0(0%)	1(6%)	2(2%)
Living arrangements	Lives alone	10(20%)	2(13%)	2(12%)	14(17%)
	Lives with carer	36(72%)	13(87%)	14(82%)	63(77%)
	Other	4(8%)	0(0%)	1(6%)	5(6%)
Baseline symptom and quality of life assessments Mean (Mean, SD)	EQ-5D	0.47(0.59, 0.32)	0.31(0.27, 0.40)	0.29(0.26, 0.35)	0.40(0.52, 0.35)
	EQ-5D Health Status	58.1(50.0,15.8)	49.0(50.0,17.1)	53.3(50.0,26.1)	55.9(50.0,17.8)
	POS	13.7(14.5,5.7)	11.9(10.0,6.1)	14.3(16.0,4.5)	13.5914.5,5.6)
	POS-PP	21.4(20.5,10.8)	20.4(23.0,10.0)	26.0(26.0,6.0)	21.9(24.0,10.1)
	PDQ-8	43.2(46.0,20.1)	35.8(41.5,18.4)	47.6(50.0,16.4)	42.8(46.0,19.4)
	HADS	22.6(23,4.1)	20.4(22.0,8.3)	19.4(19.5,4.2)	21.8(23.0,4.9)
Follow-up status at study end	Still alive	39(78%)	7(47%)	7(41%)	53(65%)
	Died	3(6%)	7(47%)	8(47%)	18(22%)
	Too ill	1(2%)	1(7%)	1(6%)	3(4%)
	Withdrew	4(8%)	0(0%)	1(6%)	5(6%)
	Lost to follow-up	3(6%)	0(0%)	0(0%)	3(4%)
Duration of illness since diagnosis (years)	Mean(SD)	11.9(5.8)	4.6(2.7)	5.9(4.0)	9.3(6.0)
	Median (min, max)	5.8(1.0,25.0)	2.7(1.0,11.0)	4.0(1.0,14.0)	8.0 (1.0, 25.0)

* chi-squared comparison between groups = 11.5 df = 4, p = 0.021.

Hoehn & Yahr stage meaning is.

3 Mild to moderate bilateral disease; some postural instability; physically independent.

4 Severe disability; still able to walk or stand unassisted.

5 Wheelchair-bound or bedridden unless aided.

### Baseline symptoms, quality of life and psychological problems

EQ5D and PDQ-8 scores showed poor quality of life (EQ5D mean score = 0.4, PDQ-8 = 42.8). Mean (SD) HADS-14 score was 21.6(5.0), and this did not differ between diagnoses, showing considerable psychological distress. The total mean POS score was moderate, approaching 14 out of a maximum of 40. Symptoms were highly prevalent (table 3) in all conditions. POS-PP showed patients reported a mean (SD) of 10.6(4.0) symptoms, with a mean (SD) score overall of 22.1(9.9). Four symptoms were reported in more than 80% of patients – problems using legs, fatigue/lack of energy, feeling sleepy and pain. Nine further symptoms were present in over half of patients - difficulty in communicating, mouth problems (drooling), use of arms & hands, spasm/stiffness, sleep problems, constipation, difficulty with bladder control, problems swallowing and shortness of breath.

**Table 3:** Prevalence (%) of symptoms in patients with different conditions at baseline interview.

Item	Symptoms	IPDn = 50 (61%)	PSPn = 15 (18%)	MSAn = 17 (21%)	TotalN = 82
1	Pain	86.0	60.0	88.2	81.7
2	Spasms	60.0	53.3	70.6	61.0
3	Fatigue/lack of energy	84.0	80.0	88.2	84.2
4	Shortness of breath	54.0	46.7	47.1	51.2
5	Nausea	24.0	6.7	41.2	24.4
6	Vomiting	8.0	0.0	5.9	6.1
7	Poor appetite	22.0	26.7	35.3	25.6
8	Problems swallowing	40.0	60.0	76.5	51.2
9	Mouth problems	70.0	66.7	82.4	72.0
10	Feeling sleepy	86.0	73.3	82.4	82.9
11	Difficulty in sleeping	58.0	40.0	82.4	59.8
12	Constipation	54.0	60.0	76.5	59.8
13	Difficulty with bowel control	24.0	20.0	58.8	30.5
14	Difficulty with controlling urine	52.0	46.7	70.6	54.9
15	Pressure sores	6.0	6.7	11.8	7.3
16	Problems using arms	64.0	73.3	82.4	69.5
17	Problems using legs	80.0	86.7	94.1	84.2
18	Difficulty communicating	58.0	80.0	76.5	65.9
19	Falls	36.0	53.3	47.1	41.5
20	Hallucinations	28.0	13.3	29.4	25.6

### Longitudinal trajectories

For the longitudinal data, there was attrition in all disease groups with loss of ∼36% to follow-up interviews, between baseline and 12 months for IPD, but more than 50% for MSA and PSP, despite the fact that interviews were scheduled at the homes of the patients. Some of this loss was related to deaths in the PSP and MSA groups (Table 2).

Even for those who survived but could not be interviewed, ‘missingness’ is unlikely to be random (i.e. it is likely to be associated with more severe disease). Most attrition was explicitly as a result of death (18 patients) or illness (3 patients) (table 2), although it may be that some of the 8 who withdrew or were lost to follow-up also had greater illness.

**Figure 1:**
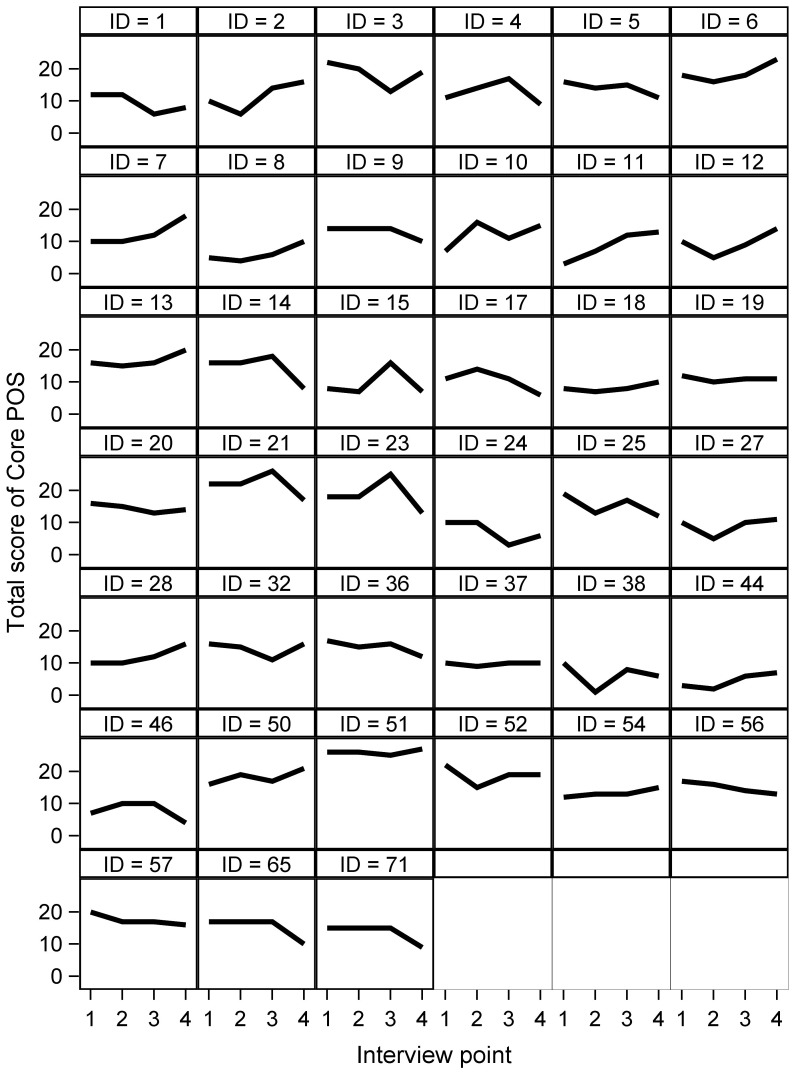
Individual trajectories of total scores of Palliative care Outcome Scale (POS). Note: Increased score equals more palliative problems/needs, only patients with 4 assessments plotted.

Examination of plots of individual patient trajectories assessed by POS, POS-PP,HADS and PDQ -8 (Figure 1, and figures S1,S2,S3) reveals that, on average, subjects who remain in the study (mostly IPD subjects, but also some people with PSP or MSA) deteriorate or remain unchanged over the study. Half of the patients showed either an upward (worsening, 24/60) or fluctuant (8/60) trajectory for POS (palliative problems), POS-PP and HADS.

### Predictors of severe outcomes

**Figure 2:**
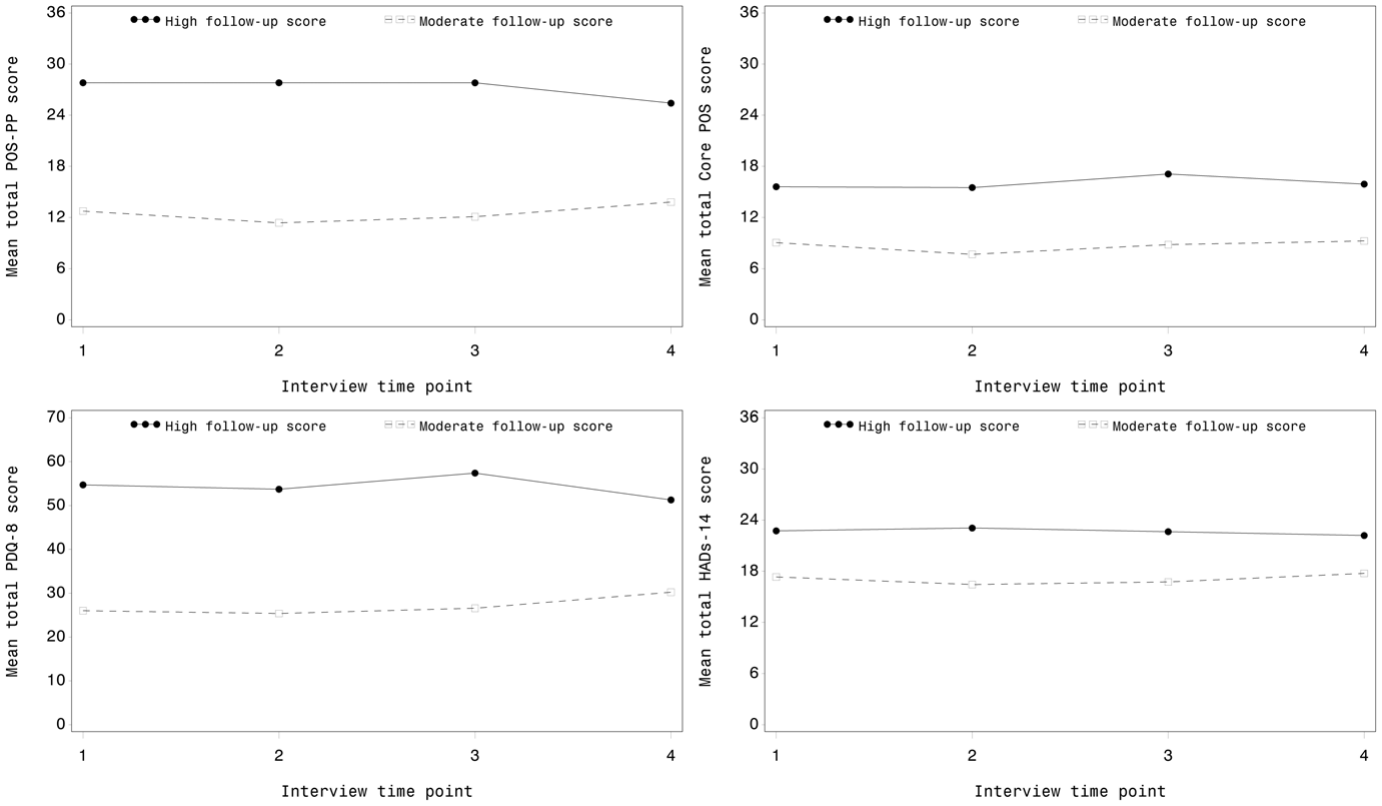
Mean total score trajectories (POS-PP, POS, PDQ-8 and HADS) by patterns of follow-up scores.

Grouping patients according to their outcome of higher or lower problems shows that those patients with severe problems at the end of the final interview also had severe problems or symptoms earlier during their illness (figure 2).

**Figure 3:**
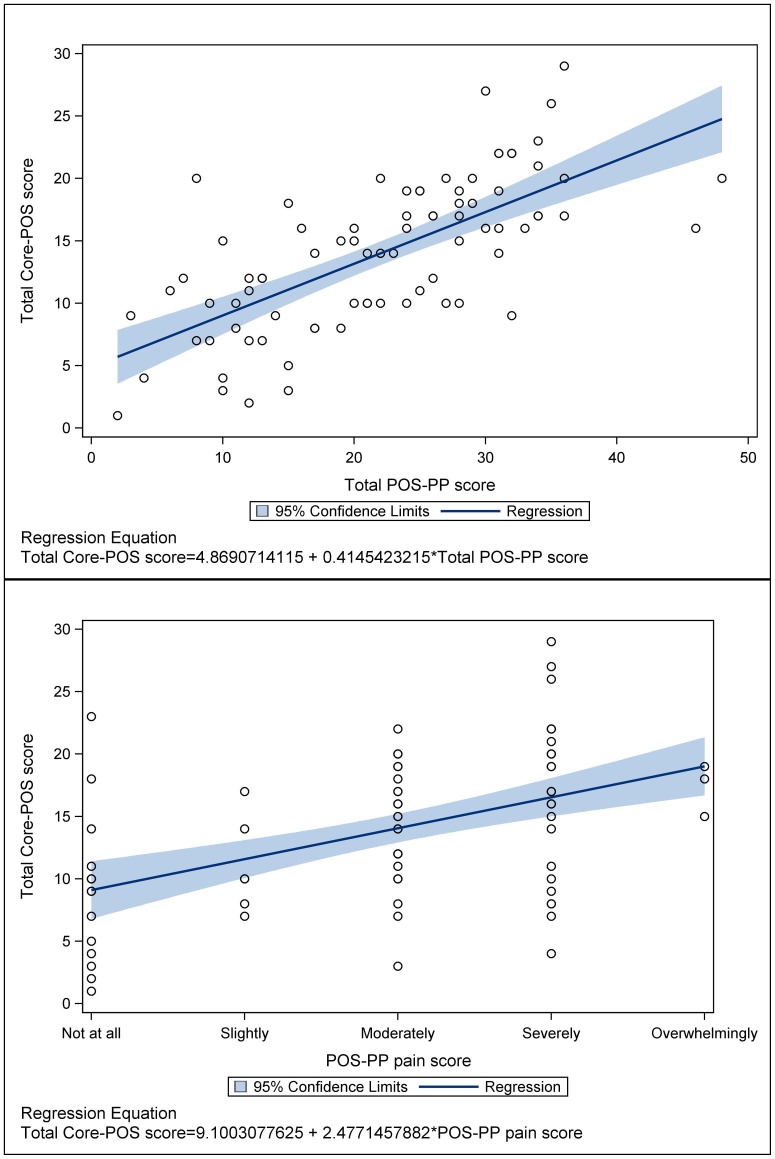
Relationship between baseline total Core-POS score and final POS-PP total score, Pain score.

Table 4 shows the results of ordinal logistic regression models. Of the demographic variables, only gender remained significant in multivariable analysis, with men having a greater chance of subsequent physical symptoms according to POS-PP(AOR: 5.18; 95%CI: 1.17 to 22.92). Gender had no effect on psychological symptoms on HADS, or palliative problems on core POS. More severe stage – as indicated by H&Y - was not significantly predictive of severe symptoms subsequently. High initial palliative problems (as indicated by POS) most consistently predicted future problems on all the outcomes, although symptoms(POS-PP), poor quality of life(PDQ-8) or psychological distress (as indicated by HADs) also showed predictive effects. Imputed data showed similar results. Figure 3 illustrates this, showing the relationship between scores on the baseline POS and final POS-PP and the single pain item.

**Table 4:** Unadjusted and adjusted risk ratios (95% CI)^a^ of factors associated with high score at follow-up time points in patients with late stage Parkinson and Parkinsonism's disease.

Predictor	Group	Outcome variable							
		POS-PP		Core-POS		PDQ-8		HADs-14^b^	
		Unadjusted	Adjusted	Unadjusted	Adjusted	Unadjusted	Adjusted	Unadjusted	Adjusted
**Baseline POS-PP**	–	**1.22** ** **(1.13 to 1.31)**	1.10(0.97 to 1.24)	**1.12** **(1.06 to 1.18)** **	**0.89** **(0.81 to 0.99)** *	**1.11** **(1.05 to 1.17)** **	**0.89** **(0.81 to 0.99)** *	1.04(0.99 to 1.10)	–
**Baseline Core-POS**	–	**1.40** **(1.23 to 1.60)** **	**1.30** **(1.05 to 1.60)** *	**1.43** **(1.25 to 1.63)** **	**1.52** **(1.24 to 1.87)** **	**1.21** **(1.09 to 1.34)** **	**1.52** **(1.24 to 1.87)** **	**1.17** **(1.05 to 1.30)** **	1.10(0.97 to 1.25)
**Baseline PDQ-8**	–	**1.09** **(1.05to 1.13)** **	**1.02** **(0.97 to 1.07)**	**1.08** **(1.05 to 1.12)** **	**1.28** **(1.09 to 1.51)** **	**1.14** **(1.08 to 1.20)** **	**1.08** **(1.03 to 1.14)** **	**1.04** **(1.01 to 1.06)** *	1.01(0.98 to 1.05)
**Baseline HADs-14**	–	**1.22** **(1.06 to 1.40)** **	**1.22** **(1.02 to 1.46)** *	**1.24** **(1.08 to 1.42)** **	**1.18** **(1.01 to 1.38)** *	**1.15** **(1.01 to 1.30)** *	1.18(1.01 to 1.38)*	**1.27** **(1.11 to 1.45)** **	**1.21** **(1.06 to 1.39)** **
**Sex**	Female vs Male	**3.20** **(1.22 o 8.35)** *	**5.19** **(1.17 to 22.95)** *	1.48(0.59 to 3.71)	–	1.16(0.45 to 2.97)	–	0.74(0.27 to 2.02)	–
**Stage**	Mild bilateral disease vs Wheel Chair	**0.16** **(0.04 to 0.56)** **	0.18(0.02 to 1.46)	0.74(0.23 to 2.40)	–	**0.23** **(0.06 to 0.81)** *	0.15(0.02 to 1.39)	3.38(0.92 to 12.39)	–
	Severe disability vs Wheel Chair	1.16(0.38 to 3.55)	1.10(0.23 to 5.23)	1.49(0.48 to 4.62)	–	0.87(0.28 to 2.77)	0.98(0.20 to 4.78)	3.12(0.87 to 11.21)	–

a : Risk ratios were estimated using the ordinal logistic regression models, the risk ratio indicates how much more likely people with a risk factor are to have higher score in the follow-up assessment, where 1 indicates no risk in comparison to the reference group. The factors used to construct the models are showing in columns corresponding to individual outcome variables.

b : Sample size n = 51, the modelling of other outcome variables were based on a sample size of 61.

Statistically significant effects (p<0.05) are in bold,

* p<0.05,

** p<0.01.

## Discussion

We found that people with late stage IPD, PSP and MSA, as shown by H&Y stage 3-5, experience profound symptoms, physical and psychological. The levels of symptoms and problems were similar to, or more severe than, those entering palliative care with cancer [18]–[21], [23], [24]. Common symptoms (affecting over 50%) were: problems using legs, fatigue/lack of energy, feeling sleepy, pain, difficulty in communicating, mouth problems (drooling), use of arms & hands, spasm/stiffness, sleep problems, constipation, difficulty with bladder control, problems swallowing and shortness of breath. Some of these symptoms, especially pain and shortness of breath, have effective treatments in cancer patients, which may need to be developed for Parkinson's syndromes. The levels of PDQ-8 and EQ5-D indicated poorer quality of life than previous samples (even the sickest subgroups) of people with Parkinson syndromes [2], [6], [9]. For the HADS, caseness of anxiety or depression is considered when either sub-scale achieves a score of greater than 8, with severe problems being considered as over 21 on the total scale. Our median score was more than this, suggesting that half of the sample had severe depression and/or anxiety. Over a third of the sample overall died during the course of the study, suggesting that we had correctly identified those with late stage disease. Survival was poorer for people with MSA and PSP validating our diagnosis of these patients and pointing to their more urgent needs. MSA and PSP are rarely studied, as is stage 5 IPD which is frequently omitted in clinical studies, thus the survival and symptom data for these groups is especially valuable.

Over the course of one year, half of all patients deteriorated in terms of symptom control and psychological or palliative distress. Analyses of final symptom and quality of life assessments, disappointingly, show that those with initial severe symptoms continued to experience these. The initial scores on the Palliative care Outcome Scale (POS) and the PDQ-8 predicted problems later.

Given the long term nature of the symptoms and the different courses, traditional models of palliative care used in cancer may not be appropriate. However, in the USA a much higher proportion of non cancer patients are referred to hospice care than in the UK [25], suggesting that here at least solutions have been found for non-cancer patients. Older patients and those from lower socio-economic groups have been shown to miss out on palliative care, with lower levels of health care spending [26]. This would apply to many of those in our study. There are urgent needs to address in the care for people with neurological diseases approaching the end of life, including dementia. The prevalence of these conditions is growing rapidly in our ageing populations. Older people face particular challenges if they are to age and die well in the place and manner of their choosing, especially those who life alone [27], as was the case for 1 in 5 of our sample. Mitchell et al highlight the need to design and test interventions that promote high-quality, goal-directed care [28]. A new short-term model of palliative care integrated with neurology, provided earlier in the course of illness, has been tested in UK patients with Multiple Sclerosis, and appeared to improve symptoms, reduce caregiver burden and reduce costs by preventing hospital admission [29], [30]. While neurologists may be reluctant to introduce the term ‘Palliative care’ to patients, in Germany a recent survey of 573 patients severely affected by multiple sclerosis found that 64% wanted disease progression and death and dying to be addressed by their doctor [31]. Doctors who were retrospectively viewed as avoiding raising critical aspects of the illness were perceived as less empathetic [31]. ‘Short term’ palliative care could undertake assessment, and address symptoms, psychological needs, end of life decision making and care co-ordination using specialist palliative care staff, integrated with neurology, rehabilitation and primary care teams. The aim would be to quickly resolve problems and discharge patients after 4–6 weeks, providing education and care plans with existing services. For many patients who are deteriorating slowly this might be cost-effective, and prevent depression and subsequent admissions and improve quality of life. Those patients, especially those with multiple complex problems, whose disease is more rapidly progressing (especially those with MSA and PSP), who were approaching death, or who had continuing problems, could go on to receive continuing palliative care in the community or in hospice following existing care models. This patient group has low survival, exemplified by the high death rate in follow-up, which is already a requirement for many palliative care services for referral. Some guidance already exists on the management of common symptoms arising in end stage of neurological diseases, including treatment of breathlessness, death rattle, restlessness, pain, thirst, and depression, along with consideration of difficult decisions e.g. fluid substitution and sedation in the terminal phase [32], and these could be the subject of future research.

Our study is limited by the setting - one secondary and tertiary neurology service in the south of the UK. There may well be patients who have not been referred to neurology services, and/or who had become too ill to attend for follow-up who were missed from our study. However, using an established neurological centre, with access to neurological tests and examination, including imaging, reduces the chances of misdiagnosis of IPD, MSA and PSP. Our study may be subject to the Hawthorne effect. Ethically the researcher could not observe severe distress and not offer to help in some way. On a few occasions this happened. In addition, many staff knew that patients were being followed and may have modified their practice. Therefore, it is likely that this study underestimates the actual level of problems experienced by patients and families with Parkinson's syndromes in the community. As our level of need was high this is worrying. Although we identified factors that were associated with future events we cannot assume causality. It may be that our predictive factors were associated with other problems which actually caused the subsequent higher levels of symptoms. However, the predictors we identified will be useful to clinicians in identifying those patients who may need greater subsequent support. We excluded patients already in nursing homes, and so our findings can be extrapolated to only home care samples. Our sample size was limited, and attrition was slightly higher than planned, but this was an extremely difficult to reach group requiring home interviews due to the high levels of disability. Very little is known about the progression of advanced, particularly stage 4/5 (Hoehn and Yahr) Parkinson's, MSA and PSP. We believe our study is the first to examine longitudinal trajectories, contrasting patients with late stage IDP, PSP and MSA, and thus provides a unique contribution, highlighting potential service developments and clinical indicators to identify those with greatest future need.

### Conclusion

We correctly identified and studied a group of patients with late stage disease missed in earlier research, and were able to study them over time. Our findings of severe and continuing symptoms, psychological and palliative distress suggest that current care is not meeting patients' needs, or controlling symptoms. The severity of their problems, similar to those of palliative care cancer patients, suggests that palliative care is not being equitably offered to all those in need. While some symptoms, such as fatigue, are especially difficult to manage, other symptoms, such as pain control, have growing possibilities for management in both cancer and non cancer conditions. The Palliative care Outcome Scale (POS) or the PDQ-8 baseline score in people with H&Y of 3 or more, and/or a diagnosis of MSA or PSP could help to trigger referral for increased support and palliative care. Research is urgently needed to test symptomatic treatments and psychological support for patients with late stage Parkinson's Disease and related motor disorders.

## Supporting Information

Figure S1 **Individual trajectories of total score of POS-PP over the interview period. Note: Increased score equals more symptoms.**
(TIF)

Figure S2 **Individual trajectories of total score of HADs-14 over the interview period. Note: Increased score equals greater psychological distress.**
(TIF)

Figure S3 **Individual trajectories of total score of PDQ-8 over the interview period. Note: Increased score equals poorer quality of life.**
(TIF)
