# Supplementary figures and images for: Symptoms and Quality of Life in Late Stage Parkinson Syndromes: A Longitudinal Community Study of Predictive Factors
Source: PLoS One. 2012 Nov 7;7(11):e46327. doi: 10.1371/journal.pone.0046327 (PMC3492372; doi:10.1371/journal.pone.0046327)

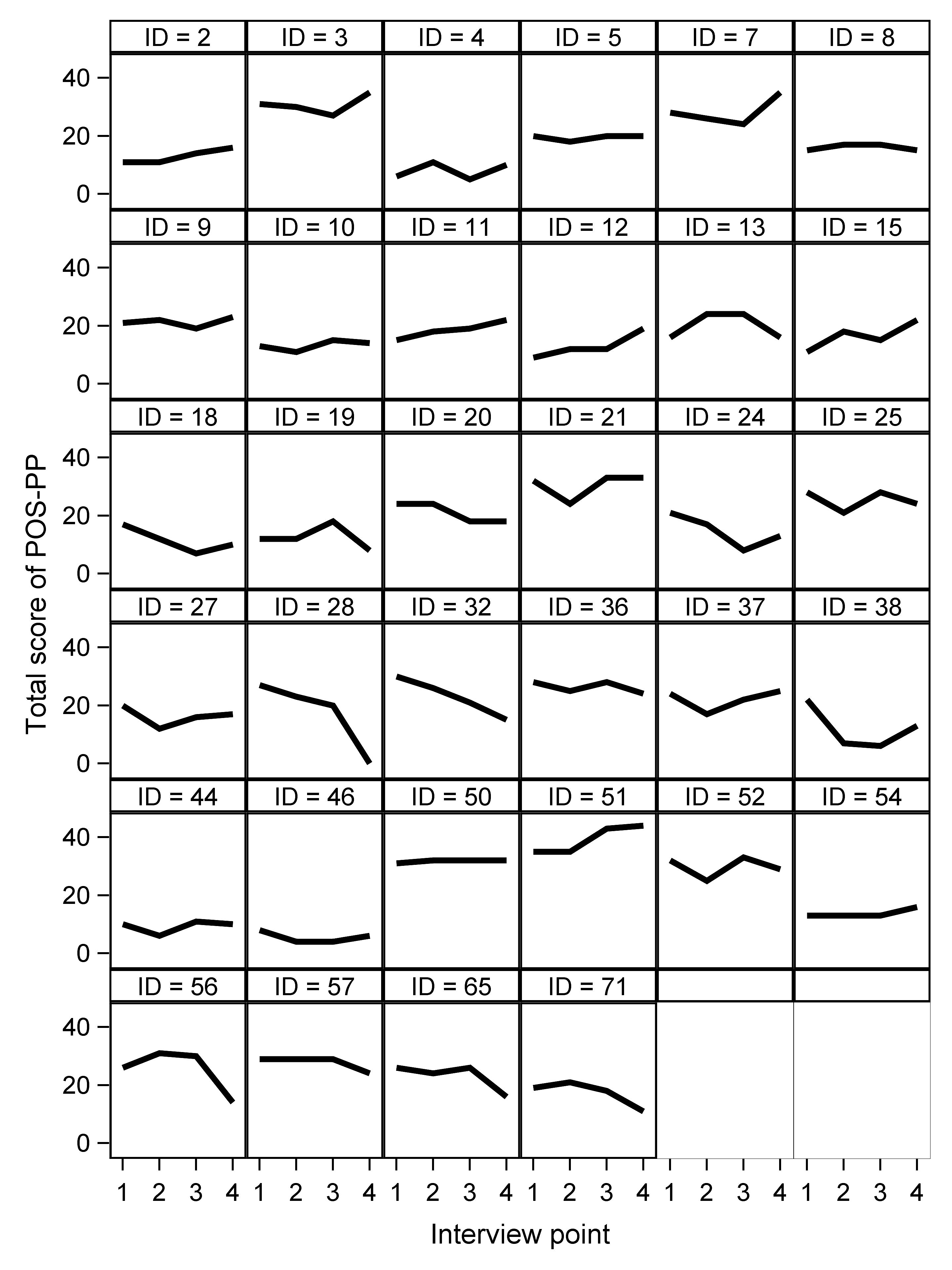

Supplement: Figure S1 — Individual trajectories of total score of POS-PP over the interview period. Note: Increased score equals more symptoms. (TIF) [file pone.0046327.s001.tif]

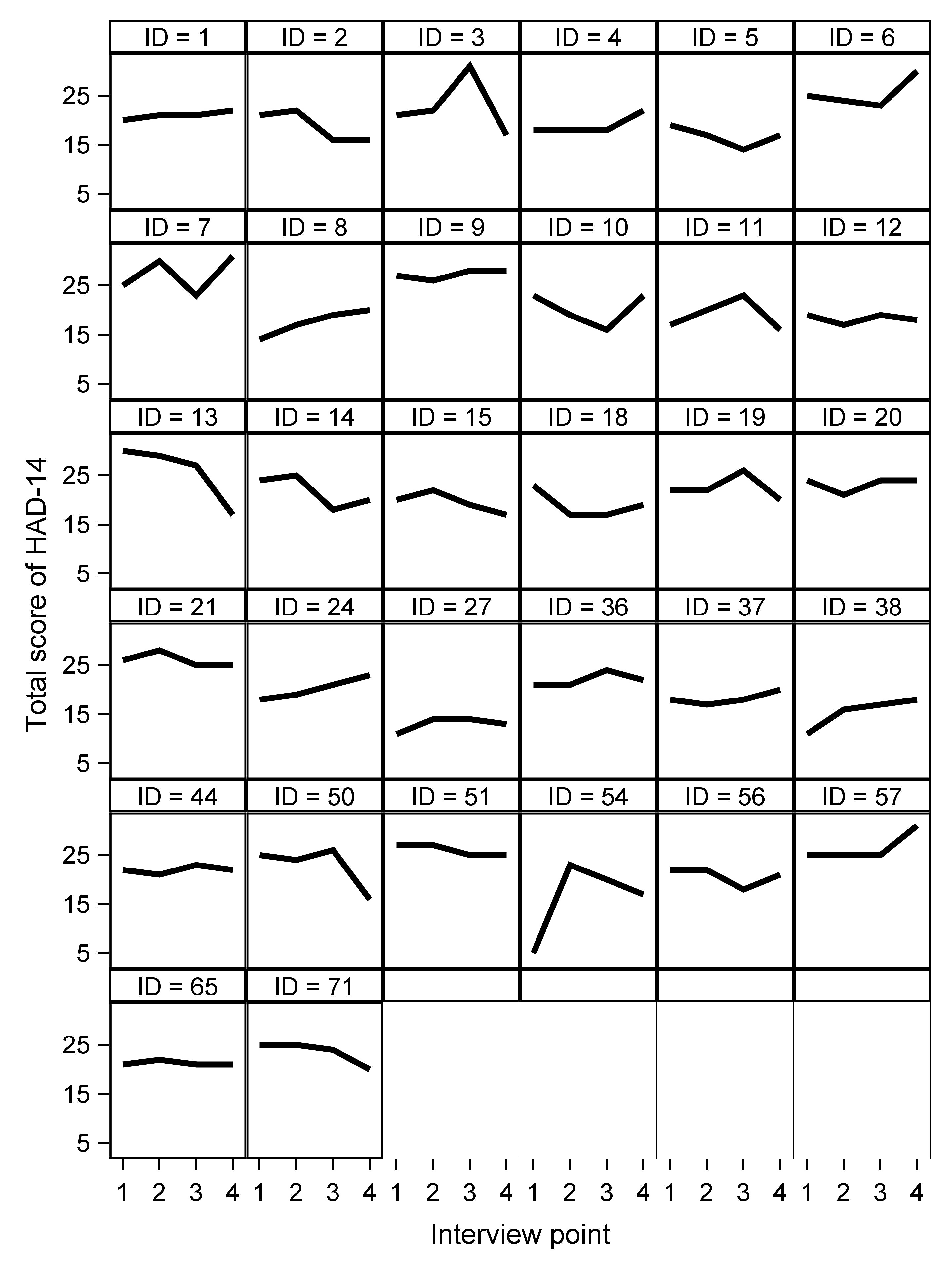

Supplement: Figure S2 — Individual trajectories of total score of HADs-14 over the interview period. Note: Increased score equals greater psychological distress. (TIF) [file pone.0046327.s002.tif]

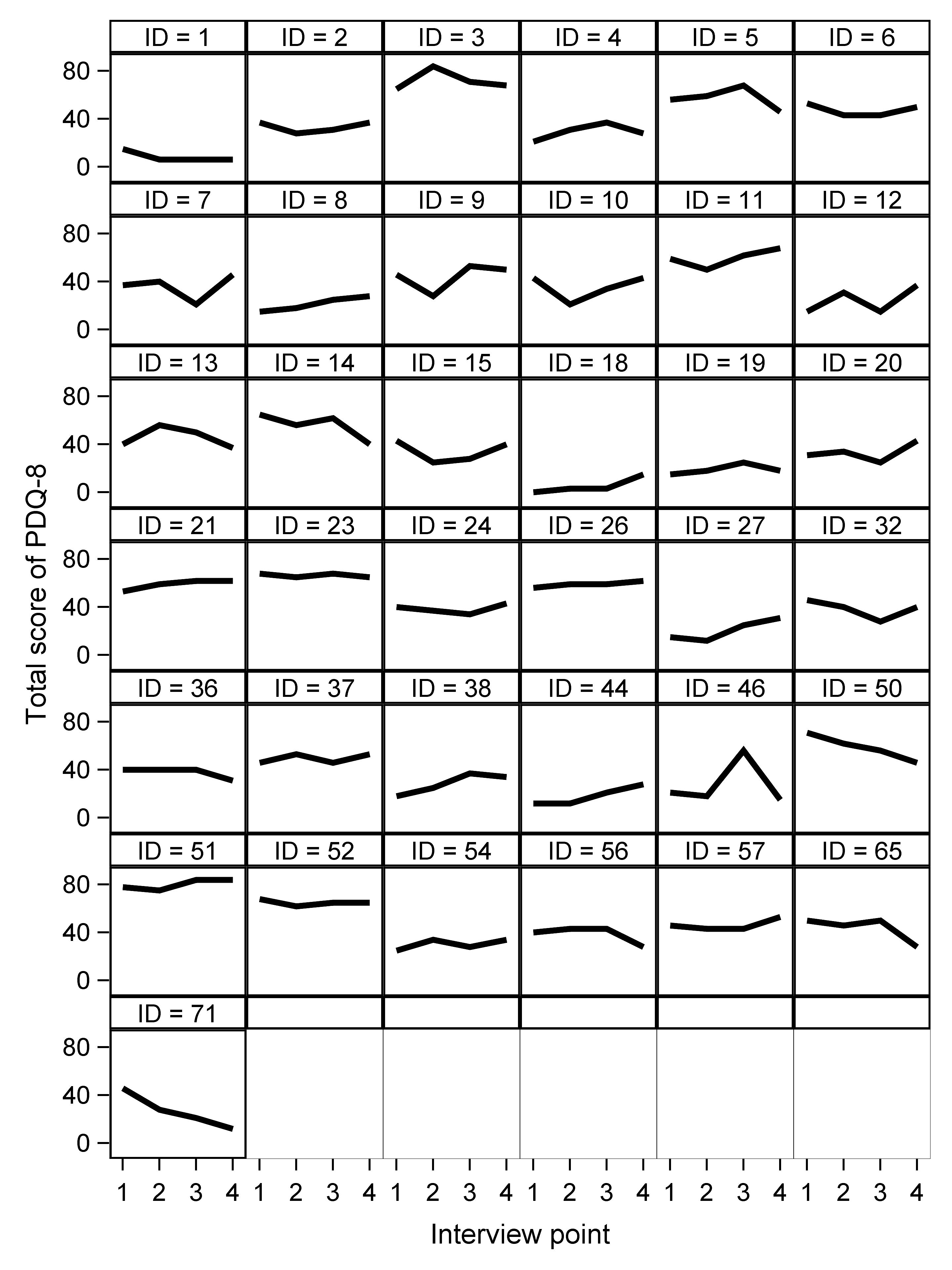

Supplement: Figure S3 — Individual trajectories of total score of PDQ-8 over the interview period. Note: Increased score equals poorer quality of life. (TIF) [file pone.0046327.s003.tif]
